# Comparison of Correlates of Bone Mineral Density in Individuals Adhering to Lacto-Ovo, Vegan, or Omnivore Diets: A Cross-Sectional Investigation

**DOI:** 10.3390/nu7053416

**Published:** 2015-05-11

**Authors:** Jessica R. Knurick, Carol S. Johnston, Sarah J. Wherry, Izayadeth Aguayo

**Affiliations:** 1 School of Nutrition and Health Promotion, Arizona State University, 500 N. 3rd Street, Phoenix, AZ 85004, USA; E-Mail: jessica.knurick@asu.edu; 2 Department of Medicine, University of Colorado, 12631 East 17th Avenue, Aurora, CO 80045, USA; E-Mail: sarah.wherry@ucdenver.edu; 3 The University of Arizona College of Medicine-Phoenix, 550 E. Van Buren St., Phoenix, AZ 85004, USA; E-Mail: iaguayo@email.arizona.edu

**Keywords:** vegetarian, vegan, bone mineral density, protein, acid-base balance

## Abstract

Vegetarian diets are associated with factors that may not support bone health, such as low body mass and low intakes of protein; yet, these diets are alkaline, a factor that favors bone mineral density (BMD). This study compared the correlates of BMD in young, non-obese adults consuming meat-based (*n =* 27), lacto-ovo vegetarian (*n =* 27), or vegan (*n =* 28) diets for ≥1 year. A 24 h diet recall, whole body DXA scan, 24 h urine specimen, and fasting blood sample were collected from participants. BMD did not differ significantly between groups. Protein intake was reduced ~30% in individuals consuming lacto-ovo and vegan diets as compared to those consuming meat-based diets (68 ± 24, 69 ± 29, and 97 ± 47 g/day respectively, *p =* 0.006); yet dietary protein was only associated with BMD for those following vegan diets. Urinary pH was more alkaline in the lacto-ovo and vegan groups *versus* omnivores (6.5 ± 0.4, 6.7 ± 0.4, and 6.2 ± 0.4 respectively, *p =* 0.003); yet urinary pH was associated with BMD in omnivores only. These data suggest that plant-based diets are not detrimental to bone in young adults. Moreover, diet prescriptions for bone health may vary among diet groups: increased fruit and vegetable intake for individuals with high meat intakes and increased plant protein intake for individuals who follow a vegetarian diet plan.

## 1. Introduction

The adoption of a vegetarian diet has become increasingly popular in light of expert recommendations to follow plant-based diets for improved health outcomes. Current estimates suggest that between 3% and 5% of the U.S. population follows some variation of a vegetarian diet [1]. Vegetarianism is associated with health benefits including lower rates of obesity, diabetes, hypertension, cardiovascular disease, and some cancers [2,3]. However, the restrictive nature of the diet has prompted concern regarding possible nutrient deficiencies and a heightened risk for osteoporosis [4].

Bone is a dynamic and metabolically active tissue that requires adequate nutrients for bone modeling and mineralization throughout the life cycle. While vegetarianism is associated with several factors that may not support bone health, such as low body mass index (BMI) and low intakes of calcium, vitamins D and B12, and protein, these diets are high in nutrients that promote bone health, including magnesium, potassium, vitamins C and K, and the *n*-3 fatty acids [5,6]. Vegetarian diets are also more alkaline than omnivorous diets, a factor that favors higher bone mineral density [7,8,9]. Furthermore, the concern that vegetarian diets may adversely affect bone mineral density (BMD) and fracture risk may be conjectural as findings from several prospective longitudinal investigations did not reveal harmful effects of vegetarianism on bone health [10,11]. A 2009 meta-analysis (nine studies totaling 2749 subjects of which 68% were female) concluded that BMD was 4% lower in vegetarians as compared to non-vegetarians [12]. The authors inferred that fracture risk was ~10% greater for vegetarians *versus* non-vegetarians but concluded that this risk was clinically insignificant [12].

Hence, it is possible that bone-healthy components of vegetarian diets counterbalance the negative effects of a low BMI and low intakes of protein and calcium. The aim of this study was to investigate the associations between indicators of bone health and BMD in young, healthy, non-obese, sedentary adults adhering to meat-based, lacto-ovo vegetarian, or vegan diet.

## 2. Experimental Section

### 2.1. Participants and Diet Classification

Nonsmoking participants were recruited through university list serves, local vegetarian societies, and word-of-mouth. Based on responses to an online survey, volunteers were recruited using the following criteria: age 19–50 year, body mass index (BMI; in kg/m^2^) < 30, respective diet adherence > 1 year, and absence of any known chronic diseases. Additionally, volunteers were excluded if they were competitive athletes or training for an endurance event, were taking prescribed medications (other than oral contraception), and/or reported (if female) an irregular menstrual cycle or history of pregnancy within the preceding six months. The study was approved by the Arizona State University Institutional Review Board, and all participants provided written informed consent.

The classification of diet group and duration of adherence was assessed by the following four questions: (1) “How many times per week do you consume meat, fish, or poultry?” (2) “How many times per week do you consume dairy products (milk, cheese, butter, yogurt)?” (3) How many eggs do you consume per week?” (4) “How long have you been following your current diet?” Subjects who reported consuming at least three servings of meat, fish, or poultry per week were considered omnivores. Those who consumed between zero and three servings were excluded from the study. Individuals who reported never eating meat, fish, or poultry, but consumed at least three servings of dairy and/or three eggs per week were considered lacto-ovo vegetarians. Individuals who reported never consuming meat, fish, or dairy and never consuming dairy or eggs were considered vegan. All subjects reported following their current diet for at least one year. Physical activity levels were estimated by validated questionnaire and reported as metabolic equivalents (METS) [13].

### 2.2. Study Design

Participants visited the test site on two occasions. At the first visit, subjects completed a questionnaire that included details on age, gender, smoking status, medical history, and activity level. A 24 h diet recall was conducted by a trained nutritionist who also completed the nutritional analysis (Food Processor version 7.71; ESHA Research, Salem, OR). Fortified food products commonly consumed by vegetarian populations were manually entered if necessary to be properly accounted in the diet analyses. Diet quality was assessed by the validated REAPS scoring system [14]. Potential renal acid load (PRAL) was estimated from dietary intake using the equation: (0.49 protein (g)) + (0.037 P (mg)) − (0.021 K (mg)) − (0.026∙Mg (mg)) − (0.013 Ca (mg)) [15]. Anthropometric measurements including height, body weight, and waist circumference were obtained, as well as body fat percentage and total body bone mineral density using dual-energy X-ray absorptiometry (DXA; Lunar iDXA, General Electric Company, East Cleveland, OH, USA). Participants collected a 24 h urine sample the day prior to the second visit using the following instructions: discard the first morning void but collect all subsequent urine voids for the day including the first urine void of the second morning. Participants were provided with urine containers and collection vessels.

Participants returned to the test site for the second visit within one week in a fasted state (no food or beverage with the exception of water for 12 h). Urine samples were turned in, and a venous blood sample was collected. Urinary calcium, measured photometrically, and the blood anion gap were measured at Sonora Quest Laboratories (Tempe, AZ, USA). Urinary pH was determined using a pH meter (WTW Chekmite pH-20 Sensor, Nova pH, Woburn, MA, USA).

### 2.3. Statistical Analyses

Data are reported as mean ± SD; statistical analyses were performed using SPSS for WINDOWS (version 21; SPSS Inc., Chicago, IL, USA). Differences between means were assessed using univariate analyses controlling for age, which differed significantly between diet groups, BMI, and gender. Non-normal data were transformed prior to analysis, and, if transformation did not normalize the data, the Kruskal Wallis test was used. Nominal data were compared using Chi Square analysis. Pearson’s correlation was used to identify relations between variables controlling for age, BMI, and gender. Significance was set at *p* ≤ 0.05.

## 3. Results

A total of 409 individuals completed the online survey, and 82 qualifying individuals agreed to enroll in the study. Based on self-reported diet habits, participants were grouped into three diet groups: omnivore (*n =* 27), lacto-ovo vegetarian (*n =* 27), and vegan (*n =* 28). Participant retention was 100%; however, one participant in the lacto-ovo vegetarian group did not complete the 24 h diet recall.

Individuals adhering to a vegan diet were significantly older than individuals adhering to a meat-based diet (33.9 ± 8.6 year *versus* 27.2 ± 6.7 year respectively); whereas, the mean age of lacto-ovo vegetarians did not vary significantly from the other diet groups (31.1 ± 9.1 year) (Table 1). Anthropometric measures and physical activity levels did not differ significantly between diet groups, but BMI tended to be higher for individuals adhering to a meat-based diet as compared to individuals adhering to lacto-ovo or vegan diets (23.5 ± 3.1 (range, 18.7–29.0), 22.4 ± 2.7 (range, 18.3–28.1), and 22.3 ± 2.6 (range, 18.9–28.0) kg/m^2^ respectively, *p =* 0.092). Diet quality was superior for individuals adhering to a vegan diet as compared to the other diet groups (Table 1).

**Table 1:** Participant characteristics by diet group (omnivorous, OMN; lacto-ovo vegetarian, LOV; vegan, VEGAN) ^1^.

	OMN	LOV	VEGAN	*p*
Gender, M/F (%F)	8/19 (70)	6/21 (78)	10/18 (64)	0.546
Age, year	27.2 ± 6.7 ^a^	31.1 ± 9.1 ^a,b^	33.9 ± 8.6 ^b^	0.013
Body weight, Kg	66.8 ± 12.0	62.9 ± 10.0	64.6 ± 12.0	0.218
Height, cm	168.0 ± 10.5	167.1 ± 7.4	169.3 ± 8.9	0.716
BMI, Kg/m^2^	23.5 ± 3.1	22.4 ± 2.7	22.3 ± 2.6	0.092
Waist, cm	80.5 ± 10.6	79.8 ± 9.8	80.1 ± 9.6	0.615
Body fat,%	29.1 ± 8.8	31.7 ± 7.6	28.8 ± 7.3	0.269
Visceral fat, cm^3^	246.0 ± 238.0	254.1 ± 298.2	435.7 ± 428.4	0.260
METS, kcal kg^−1^ week^−1^	50.2 ± 39.0	37.4 ± 28.8	40.1 ± 27.5	0.569
Diet quality, REAPS score	31.8 ± 3.2 ^a^	32.4 ± 2.7 ^a^	36.1 ± 2.0 ^b^	<0.001

^1^ *p* for univariate analyses except gender where *p* represents Chi Square analysis (non-normal data transformed prior to analysis (age and visceral fat)); means with different superscripts differ significantly *p* < 0.05. Anthropometric data, METS, and diet quality analyses adjusted for age.

BMD was reduced 4%–5% in individuals adhering to meatless diets as compared to the omnivores; however, this difference was not significant (Table 2). Z scores did not differ significantly between diet groups and placed BMD between the 75th and 80th percentiles for meat-eaters and between the 60th and 70th percentiles for vegetarians. Calcium excretion (mg/24 h) was significantly higher (~34%) in the omnivores as compared to individuals adhering to vegetarian diets (*p =* 0.045). Several acid-base indices (urinary pH and dietary PRAL) varied significantly between diet groups. Urinary pH was more alkaline in the vegetarian diet groups *versus* the omnivores (6.5 ± 0.4 and 6.7 ± 0.4 for the lacto-ovo and vegan groups *versus* 6.2 ± 0.4 for the omnivores, *p =* 0.003). Dietary PRAL was reduced over 100% in the vegetarian groups as compared to the omnivores (*p =* 0.001; Table 2). PRAL was significantly related to urinary pH among all participants (*r* = −0.322, *p =* 0.004).

The intake of several nutrients commonly associated with bone health varied by diet group (Table 3). Protein intake was reduced ~30% in individuals adhering to vegetarian diets as compared to those consuming a meat-based diet (68 ± 24 and 69 ± 29 g/day for individuals adhering to lacto-ovo or vegan diets *versus* 97 ± 47 g/day for individuals eating a meat-based diet, *p =* 0.006). Additionally, individuals adhering to vegan diets consumed significantly more magnesium, folate, and vitamin K as compared to their omnivorous counterparts (Table 3). However, vitamin B12 intakes were significantly reduced in the vegetarian diets *versus* the meat-based diet. Dietary protein was significantly correlated with both BMD and urinary calcium among all participants (*r* = 0.274, *p =* 0.017 and *r* = 0.228, *p =* 0.049 respectively). However, when examined by diet group, dietary protein was significantly related to BMD only in the vegan diet group (Table 4); moreover, dietary protein was not associated with urinary calcium in any single diet group. No other significant associations were noted for individual nutrients and BMD for any diet group. Urinary pH was the only other variable significantly related to BMD when examined by diet group, an association only noted in the omnivore diet group (*r* = 0.602, *p =* 0.002; Table 4). There was no association between urinary pH and BMD in the sample as a whole.

**Table 2:** Bone mineral density and urinary measures by diet group (omnivorous, OMN; lacto-ovo vegetarian, LOV; vegan, VEGAN) ^1^.

	OMN (*n =* 27)	LOV (*n =* 27)	VEGAN (*n =* 28)	*p*
Bone mineral density, g/cm^3^	1.18 ± 0.11	1.12 ± 0.10	1.13 ± 0.11	0.384
*T* score	0.71 ± 0.97	0.13 ± 0.93	0.03 ± 0.85	0.286
*Z* score	0.77 ± 0.77	0.42 ± 0.75	0.26 ± 0.86	0.337
Urinary calcium, mg/24 h	155 ± 71 ^a^	115 ± 64 ^b^	117 ± 65 ^b^	0.045
Anion Gap	12.5 ± 2.6	11.9 ± 2.0	11.9 ± 2.4	0.562
Urinary pH	6.2 ± 0.4 ^a^	6.5 ± 0.4 ^b^	6.7 ± 0.4 ^b^	0.003
PRAL	19.6 ± 24.3 ^a^	−1.5 ± 23.9 ^b^	−15.2 ± 40.5 ^b^	0.001 ^2^

^1^ *p* for univariate analyses analysis (non-normal data transformed prior to analysis (urinary calcium, anion gap)); all *p* values adjusted for BMI, age, and gender; means with different superscripts differ significantly *p* < 0.05. Sample reduced for PRAL due to a missing diet recall (*n =* 25, 26, and 28 for OMN, LOV and VEGAN respectively). ^2^ Non parametric Kruskal-Wallis test.

## 4. Discussion

Bone is sensitive to its environment and slight changes in nutrient availability and acid-base balance have acute effects on bone metabolism, which in the long term would theoretically impact BMD [16,17]. BMD did not differ between lacto-ovo vegetarians, vegans, and omnivores in the present study supporting the contention that plant-based diets are not detrimental to bone in young adults. Vegetarian diets, particularly vegan diets, have a nutrient profile that varies significantly from meat-based diets, and many of the nutrient differences could theoretically impact bone metabolism, some favorably and some unfavorably. In young adults, a well-balanced vegetarian diet may provide adequate bone-enhancing properties that negate the diet properties that adversely impact bone mineralization. However, these data cannot provide insights regarding the impact of vegetarian diets on bone health in older adults or the elderly.

**Table 3:** Dietary intake for nutrients associated with bone health by diet group (omnivorous, OMN; lacto-ovo vegetarian, LOV; vegan, VEGAN) ^1^.

	OMN (*n =* 27)	LOV (*n =* 26)	VEGAN (*n =* 28)	*P*
Energy, kcal	2108 ± 727	2042 ± 558	2069 ± 665	0.856
Protein, g	97 ± 47 ^a^	68 ± 24 ^b^	69 ± 29 ^b^	0.006
Calcium, mg	939 ± 516	746 ± 422	768 ± 415	0.241
Magnesium, mg	210 ± 144 ^a^	241 ± 135 ^a^	354 ± 183 ^b^	0.006
Potassium, mg	2048 ± 1153	2135 ± 1220	2876 ± 1934	0.154
Sodium, mg	3743 ± 1877	2871 ± 1389	2522 ± 1228	0.067
Zinc, mg	8.7 ± 8.2	5.6 ± 3.8	8.5 ± 6.1	0.210
Folate, ug	286 ± 233 ^a^	391 ± 290 ^ab^	549 ± 367 ^b^	0.006
Vitamin B6, mg	1.5 ± 1.0	2.5 ± 4.2	2.4 ± 2.2	0.256 ^2^
Vitamin B12, mg	4.9 ± 8.0 ^a^	2.3 ± 3.4 ^b^	3.3 ± 5.1 ^b^	0.051 ^2^
Vitamin C, mg	149 ± 134	140 ± 128	225 ± 177	0.143
Vitamin D, ug	2.2 ± 2.9	1.3 ± 2.5	1.7 ± 2.3	0.089 ^2^
Vitamin K, ug	100 ± 200 ^a^	159 ± 251 ^ab^	557 ± 871 ^b^	0.015
*n*-6 fatty acids, g	6.3 ± 7.4	5.8 ± 5.8	4.0 ± 3.1	0.479 ^2^
*n*-3 fatty acids, g	0.8 ± 0.8	0.7 ± 1.0	1.3 ± 2.2	0.626 ^2^
*n*-6/*n*-3 ratio	8.8 ± 5.3 ^ab^	10.8 ± 7.3 ^a^	6.4 ± 5.4 ^b^	0.026

^1^ *p* for univariate analyses analysis (transformed prior to analysis: protein, calcium, magnesium, potassium, sodium, zinc, folate, vitamin K, *n*-6 and *n*-3 fatty acids); all *p* values adjusted for BMI, age, and gender; means with different superscripts differ significantly *p* < 0.05. ^2^ Non parametric Kruskal-Wallis test.

**Table 4:** Correlation coefficients for urinary pH and protein significantly correlated with BMD in at least one diet group ^1^.

	OMN	LOV	VEGAN
Urinary pH	0.602 *	0.003	−0.008
Protein, g	0.190	0.262	0.434 *

^1^ Asterisk denotes a significant correlation: Pearson’s Correlation, *p* < 0.05.

Much evidence demonstrates the importance of dietary protein for bone accrual and reduced fracture risk particularly in older adults; hence, this nutrient is of particular concern since vegetarian diets are comparatively low in protein. The anabolic influence of protein on bone is mediated by insulin-like growth factor-1, which increases plasma osteocalcin and promotes osteoblast recruitment and activity [18]. Elderly patients with a recent osteoporotic hip fracture ingesting a protein supplement (20 g/day) experienced marked elevations in insulin-like growth factor-1 (+84%) and significant attenuation of proximal femur bone loss as compared to their control counterparts after six months [19]. Dietary protein also promotes bone mineralization by enhancing calcium absorption rates; however, adequate levels of dietary calcium (≥1000 mg/day) are likely necessary for this effect [20]. A cohort study based in California with 1865 female participants (≥45 year at study start) and a 25 year follow-up reported a significantly higher incidence of wrist fracture in vegetarians as compared to omnivores and that higher intakes of vegetable protein by vegetarians, from <3 × weekly to >1 × daily, reduced this risk by 68% [10]. Conversely, the incidence of vertebral fracture risk did not differ between vegetarians and omnivores in an Asian cohort of postmenopausal women followed for two years (incidence of fracture: 5.7% and 5.4% respectively), even though vegetarian participants consumed 40% less protein daily than their omnivore counterparts [11]. However, the daily protein intake was relatively low even for the omnivores (62 g/day), and the rates of fracture were much higher than that reported for the California cohort (3.7/1000 person-years). A two year prospective, cohort trial in female collegiate runners (22 ± 3 years) did report a significant, positive impact of animal protein intake on bone accrual, a relationship that remained significant after controlling for calcium intake [21]. However, in this study, dietary protein was not related to fracture risk.

Although important for bone anabolism, a high protein intake can adversely impact bone health as it contributes to the dietary acid load, a consequence of the hydrogen ions produced during the oxidation of the sulfur-amino acids, methionine and cysteine [22]. Since the dietary acid load stimulates osteoclast activity and bone resorption, enabling the release of neutralizing carbonate and hydroxyapatite salts from bone, bone mineralization is hindered [23,24]. The dietary acid load is estimated by measuring 24 h urinary pH, an indirect marker of net acid excretion [25], or from daily intakes of protein and phosphorus, and the alkaline salts, calcium, potassium, and magnesium, using a formula known as PRAL, the potential renal acid load [15].

Demonstrating the two-edged effect of dietary protein on bone, Thorpe and colleagues reported that although increasing dietary protein was associated with higher BMD in postmenopausal women, this favorable effect was attenuated by a high sulfur intake [26]. These results may help explain the lack of an association between dietary protein and BMD in omnivores in the present study. The inter-quartile range for dietary protein in the omnivores (69–124 g) was above the recommended protein intake for this participant sample (52 g; calculated using the U.S. Food and Nutrition Board’s dietary goal: 0.8 g/kg body weight). High intakes of protein likely contributed to the dietary acid load as indicated by the low urinary pH for omnivores; moreover, urinary pH predicted 36% of the variance in BMD in this diet group. The inter-quartile range of dietary protein in the lacto-ovo and vegan diet groups, 44–93 g and 51–87 g respectively, encompassed the dietary goal for protein, and dietary protein was not related to urinary pH in these diet groups. However, dietary protein was strongly related to BMD in the vegan diet group suggesting the importance of ample dietary protein for bone health even in a marked alkaline environment (urinary pH, 6.7 ± 0.4; PRAL, −15.2 ± 40.5).

High intakes of fruits and vegetables reduce the dietary acid load by providing neutralizing anions, e.g., citrate and malate, found mainly in association with potassium and magnesium cations, and glutamate, a neutralizing anionic amino acid [27]. Large controlled trials have demonstrated the benefits of supplemental potassium citrate on BMD in postmenopausal women with osteopenia after one year [28] and in healthy elderly men and women after two years [29]; however, Macdonald *et al*. [30] were unable to increase BMD in healthy postmenopausal women with potassium citrate supplements taken daily for two years. Plant-based diets are also high in vitamin K, an essential cofactor in the carboxylation of osteocalcin necessary for optimal mineralization of bone [31]. Vegan diets contained at least 35%, 47%, and 250% more potassium, magnesium, and vitamin K respectively than the other diet plans, a characteristic reflected by the very low PRAL score and high urinary pH of the vegan diet group. The similar BMD among the diet groups may reflect a cancelling effect occurring with the different diet plans: high dietary protein (bone positive) with high dietary acid load (bone negative) in the omnivore group but low dietary protein (bone negative) with low dietary acid load (bone positive) for the vegetarian groups. A healthy bone diet prescription may be to increase fruit and vegetable intake among individuals with high meat intakes and to increase plant protein intake among individuals who follow a vegetarian diet plan.

Gunn and colleagues [32] examined the feasibility of increasing fruit and vegetable consumption in 21 healthy omnivorous adult women (40–65 year of age; typical fruit and vegetable intake <5 servings/day) in order to reduce the renal acid load as indicated by an increase in urinary pH. After the 8-week intervention, participants increased fruit consumption by 1.4 servings/day and vegetable consumption by 2.3 servings/day; a non-significant reduction in dietary protein was also noted (80 g at baseline and 73 g at week 8; *p =* 0.29). Importantly, urinary pH increased significantly, from 5.46 at baseline to 6.14 at week 8. Intervention studies are needed to determine if a change in acid-base balance due to increased fruit and vegetable consumption favors a bone formation environment in omnivores. To date, the effect of increased protein intakes on bone health in vegetarians have not been conducted; yet, the results from several intervention trials suggest a benefit of supplemental soy protein for improving muscle mass and strength in older men consuming a vegetarian diet plan during 12 weeks of resistive training [33,34]. However, care may be needed when recommending higher protein intakes for vegetarians since some popular vegetarian protein sources, e.g., hard cheeses, eggs, brown rice, and rolled oats, have PRAL scores similar to, or greater than, lean beef and turkey (≥7.8) [35].

### Limitations of the Study

The results of this trial only suggest possible influences of omnivorous and vegetarian diet plans on BMD due to the cross-sectional study design and to the small sample sizes by diet group. Also, the placement of participants into diet groups based on self-reported dietary intakes is a major study limitation as are the narrow age range of participants and the lack of site-specific measures of BMD. Single-day diet analyses cannot account for the daily intraindividual variation in food intake over time, which limits dietary data interpretation. However, the 24 h diet recalls were performed by a trained nutritionist and carefully conducted as indicated by energy intakes that matched energy expenditure prediction equations within a 10% margin. Similarly, a random 24 h urine collection may not capture daily intraindividual variation in pH, but a 24 h urine sample, as compared to a fasting urine sample, will control for the diurnal variation in urine pH [36]. Trial strengths include the 100% retention rate, and the use of DXA for total body BMD determination and 24-h urine samples for the measurement of urinary pH. Participants were also carefully selected to reduce confounding by known predictors of BMD: physical activity, obesity, and smoking [37,38].

## 5. Conclusions

These data suggest that, in the short-term and in a small specific population, plant-based diets are not detrimental to bone in young adults. Moreover, diet prescriptions for bone health may vary among diet groups: increased fruit and vegetable intake for individuals with high meat intakes and increased plant protein intake for individuals who follow a vegetarian diet plan.
